# The effectiveness of an oral gonadotropin-releasing hormone antagonist relugolix for retained products of conception (RPOC) formed after abortion at less than 22 weeks of gestation: a retrospective study

**DOI:** 10.3389/fmed.2025.1543272

**Published:** 2025-05-27

**Authors:** Satoko Sasatsu, Yosuke Ono, Dai Miyashita, Tatsuya Yoshihara, Kota Tanaka, So Owada, Kana Makino, Akiko Nakagomi, Maki Ogi, Eriko Ogasawara, Hikaru Tagaya, Hiroko Fukasawa, Yasuhiko Okuda, Osamu Yoshino

**Affiliations:** Department of Obstetrics and Gynecology, University of Yamanashi, Yamanashi, Japan

**Keywords:** GnRH antagonist, retained products of conception, surgical intervention, miscarriage, menstrual resumption

## Abstract

**Purpose:**

This study investigated the effectiveness of an oral GnRH antagonist for retained products of conception (RPOC) carrying a risk of heavy bleeding.

**Methods:**

With IRB approval and patient consent, this retrospective study included 97 RPOC cases after miscarriage. Clinical courses of the GnRH antagonist group (*n* = 20) were compared with those of non-GnRH antagonist group (historical control, *n* = 77). Surgical intervention was performed if there is no decrease in RPOC blood flow or size after treatment initiation.

**Results:**

The reduction rate in the maximum RPOC diameter after treatment initiation in GnRH antagonist group was greater compared to that in non-GnRH antagonist group [50 (0–79.7)% vs. 15.4 (0–56.9)]%, *p* < 0.001]. The frequency of surgical intervention in GnRH antagonist group was lower (30.0%, 6/20) than that of non-GnRH antagonist group (70.1%, 54/77, *p* = 0.002). Multivariate analysis showed that GnRH antagonist reduced the risk of surgical intervention [adjusted-odds ratio (95% confidence interval); 0.20 (0.06–0.58), *p* = 0.003]. In the GnRH antagonist group, the period from RPOC diagnosis to menstrual resumption was shorter [14.5 (9–71) days] than that of non-GnRH antagonist group [26.0 (6–95) days, *p* = 0.002].

**Conclusion:**

GnRH antagonist may be a new therapeutic candidate for RPOC.

## Introduction

Retained products of conception (RPOC) refer to pregnancy tissue remaining in the uterus after pregnancy termination, including childbirth, spontaneous abortion, or induced abortion (1, 2). The incidence of RPOC is reported to be as high as 3% (3), making it a common cause of post-pregnancy bleeding, which can lead to severe hemorrhage and life-threatening risks. The increased use of assisted reproductive technology (ART) has also raised the incidence of placenta accreta, increasing the risk of RPOC after miscarriage (4). While conservative management may resolve RPOC, it often requires a prolonged period for complete resolution. When expectant management fails, surgical interventions like transcervical resection (TCR) or dilation and curettage are often needed (5–7). In cases of high hemorrhagic risk or active bleeding, interventional radiology (IVR) procedures such as uterine artery embolization (UAE) or, in extreme cases, hysterectomy, may be required (8). Both surgical interventions and IVR are associated with complications, including intrauterine adhesions, reduced pregnancy rates, and increased miscarriage rates (9, 10), making them less desirable for patients wishing to preserve fertility. For those undergoing fertility treatment, minimally invasive options are crucial for treating RPOC effectively without compromising future fertility. Controlling blood flow to RPOC is key in reducing hemorrhagic risks and may allow for less invasive procedures, even if surgical intervention is eventually required.

Recently, an oral GnRH antagonist (relugolix) has been introduced for the treatment of uterine fibroids (11). GnRH agonists can initially cause a temporary flare-up in estradiol (E2) levels, increasing the risk of bleeding (12), whereas GnRH antagonists act directly on GnRH receptors, reducing E2 levels immediately without a flare-up. We hypothesized that oral GnRH antagonists may reduce blood flow to the endometrium, including RPOC. However, limited research exists on the efficacy of GnRH antagonists for RPOC.

This study aimed to assess the effectiveness of oral GnRH antagonists by comparing the clinical outcomes of RPOC cases managed with GnRH antagonist therapy to those managed with conventional methods without GnRH antagonists.

## Patients and methods

This retrospective observational study was conducted with approval from the Ethics Committee of the University of Yamanashi Hospital (Approval Number: 2761). Written informed consent was obtained from all participants prior to inclusion. The study was conducted in accordance with the Declaration of Helsinki.

As this was a retrospective study, no interventions beyond standard clinical care were performed, and no additional risk was imposed on the patients. A total of 97 RPOC cases, following spontaneous or induced abortion at less than 22 weeks of gestation, were managed at the University of Yamanashi Hospital between January 2014 and July 2024. We selected cases from 2014 onward because surgical management standards for RPOC at our institution became more consistent during this period, minimizing potential variations in treatment strategies. RPOC was diagnosed based on the detection of intrauterine echogenic material with blood flow identified on transvaginal color Doppler ultrasonography. Blood flow was assessed according to the Gutenberg classification, and cases with Grade I or higher were considered indicative of RPOC (13). The size of RPOC was measured by transvaginal sonography, and the maximum major axis of it was used for comparison. Surgical interventions were considered if no decrease in RPOC blood flow or maximum size was observed within 4 weeks, based on evaluations conducted every 2 weeks. Patients were excluded if they had a gestational age of 22 weeks or more, a diagnosis of molar pregnancy or malignancy, significant systemic disease, or incomplete clinical follow-up. And, since 2022, we have treated those patients using oral GnRH antagonists.

**FIGURE 1:**
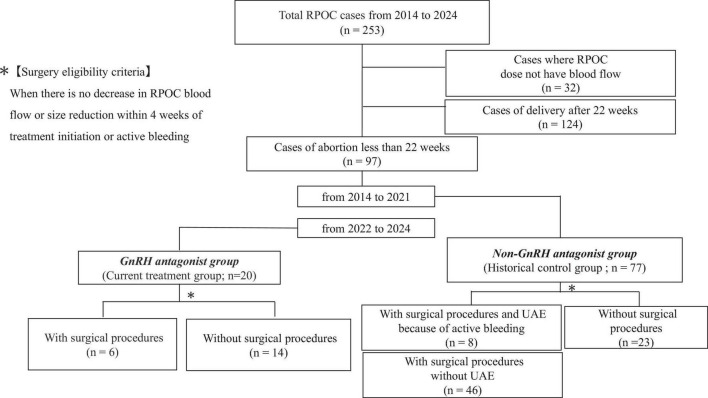
A flow chart showing how patients were enrolled in this study. RPOC; retained products of conception.

The 97 RPOC cases were divided into two groups: a GnRH antagonist group (*n* = 20) and a historical control group (*n* = 77), which did not receive GnRH antagonist treatment but had blood flow detected by color Doppler imaging (Figure 1). In the historical control group, patients were managed conservatively according to clinical judgment, which included either observation alone or surgical intervention based on clinical progression. Thus, the control group represents a standard conservative management cohort, rather than a purely expectant management group. Clinical outcomes between the two groups were compared using medical records. In 2022, oral relugolix (40 mg) was administered daily to 20 cases, and transvaginal ultrasound was used to evaluate treatment response after at least 14 days. If RPOC blood flow disappeared, treatment was discontinued, and menstruation resumption was monitored. If blood flow persisted, treatment continued for up 3–4 weeks or until the flow resolved. The reduction rate in RPOC size was calculated using the following formula:


(initialmaximumdiameter-post-treatmentmaximum

diameter)/initialmaximumdiameter×100%.

The time to menstruation resumption was defined as the number of days from the date of RPOC diagnosis to the first day of reported menstrual bleeding. These definitions were applied uniformly to all cases to ensure consistency and reproducibility of the analysis.

Clinical parameters included age, body mass index (BMI), pregnancy history, ART use, surgical intervention rates, blood transfusion rates, ultrasound findings, and menstrual information. Statistical analyses were performed using JMP version 10 (SAS Institute Inc., Cary, NC). The normality of continuous variables was assessed using the Shapiro–Wilk test. Continuous variables were compared using the Student’s *t*-test when normally distributed, and the Mann–Whitney U test when not normally distributed. Categorical variables were compared using the Chi-square test or Fisher’s exact test, as appropriate. Missing data were minimal and were handled using complete case analysis.

Multivariate logistic regression analyses were conducted using the forced-entry method to identify factors associated with surgical intervention. Three models were constructed: [Model 1: Adjusted for age and ART use; Model 2: Model 1 plus RPOC size; Model 3: Model 2 plus body mass index (BMI) and surgery for abortion]. Subgroup analysis was performed by stratifying patients by age (< 35 vs. ≥ 35 years). Sensitivity analyses were conducted to assess the robustness of the findings. Sensitivity analyses were conducted to assess the robustness of the findings. We stratified patients by ART status (natural pregnancy vs. ART pregnancy) and compared surgical intervention rates separately within each subgroup. A *p*-value < 0.05 was considered statistically significant.

## Results

The GnRH antagonist group consisted of 20 cases, 12 of which involved pregnancies achieved through ART. Among these 20 cases, RPOC occurred in 10 following spontaneous expulsion after missed abortion and in four after miscarriage surgery. Additionally, three cases followed induced abortion, and three occurred after late-term spontaneous miscarriage between 19 and 20 gestational weeks. Of the 20 cases treated with a GnRH antagonist, six required surgical intervention (Table 1).

**TABLE 1:** Clinical backgrounds of 20 cases which were administered an oral gonadotropin-releasing hormone (GnRH) antagonist.

Case	Age	G	*P*	Preceding pregnancy	Gestational age at abortion (weeks)	Artificial abortion (0 = No, 1 = Yes)	Surgery for abortion (0 = No, 1 = Yes)	Plasma hCG value at diagnosis of RPOC (mIU/mL)	RPOC diameter before taking GnRH antagonist (mm)	RPOC diameter after taking GnRH antagonist (mm)	Duration of taking GnRH antagonist (days)	Surgical procedure
1	33	3	1	ART	8	0	0	Not measured	12	12	25	TCR
2	37	1	0	ART	8	0	1	564	35	35	28	TCR
3	39	3	2	ART	9	0	0	369	22	15	7	TCR
4	32	1	0	ART	9	0	0	7.1	20	8	14	TCR
5	33	1	0	ART	8	0	0	160	12	12	53	TCR
6	40	2	0	ART	19	0	0	0.2	50	42	28	None
7	35	4	2	ART	9	0	0	144	21	6.5	20	None
8	37	1	0	ART	9	0	0	10.1	15	0	29	None
9	34	3	1	ART	14	0	1	2.6	19	18	14	None
10	27	1	0	ART	8	0	0	22	15	11.7	39	None
11	40	1	0	ART	9	0	0	23	16	7.5	40	None
12	41	0	0	ART	6	0	0	236	19	15.3	14	None
13	38	2	1	Spontaneous	11	0	1	131	23	19	28	TCR
14	39	4	3	Spontaneous	7	0	0	< 0.2	20	5.3	14	None
15	30	1	0	Spontaneous	19	0	0	< 1.2	20	19	57	None
16	22	1	0	Spontaneous	8	1	1	22	17	3	33	None
17	43	3	2	Spontaneous	11	1	1	66	59	12	97	None
18	25	3	2	Spontaneous	10	0	1	5	23	14.9	66	None
19	22	1	0	Spontaneous	9	1	1	5.5	16	12.8	22	None
20	33	1	0	Spontaneous	20	0	0	8.8	41	0	47	None

RPOC, retained products of conception; ART, assisted reproductive technology; TCR, transcervical resection.

**FIGURE 2:**
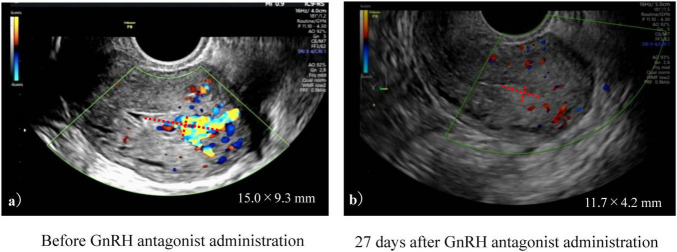
A representative imaging of retained products of conception (RPOC) in transvaginal ultrasound (Case 10, Table 1). **(a)** RPOC imaging before taking an oral Gonadotropin-Releasing Hormone (GnRH) antagonist. **(b)** RPOC imaging after taking an oral GnRH antagonist for 27 days. Red dotted lines show the long and short diameters of RPOC.

There were no significant differences between the GnRH antagonist and non-GnRH antagonist groups regarding patient background, proportion of ART pregnancies, or rate of abortion surgeries. The maximum RPOC diameter at diagnosis did not differ between the two groups (Table 2). Of the 20 patients taking GnRH antagonists, two reported mild swelling and irritability, but these symptoms resolved naturally, and no patient discontinued treatment due to side effects. In most cases, the intensity of blood flow diminished or disappeared after the start of treatment (Figure 2).

**TABLE 2:** Patient backgrounds of Gonadotropin-releasing hormone (GnRH) antagonist and non-GnRH antagonist group.

RPOC cases (*n* = 97)	GnRH antagonist (*n* = 20)	Non-GnRH antagonist (*n* = 77)	*P*-value
Age (years)	34.0 ± 6.2	33.8 ± 5.8	0.89*
Body mass index (kg/m^2^)	22.0 ± 4.3	21.1 ± 3.0	0.39*
Gravidity	1.5 (1–4)	1.5 (1–8)	0.75**
Parity	0 (0–3)	0.5 (0–4)	0.91**
Assisted reproductive technology	12 (60.0%)	35 (45.5%)	0.36***
Spontaneous abortion	16 (80%)	66 (85.7%)	0.78***
Artificial abortion	4 (20%)	11 (14.3%)	0.78***
Surgery for abortion	7 (35%)	26 (33.8%)	0.95***
Gestational age at diagnosis of abortion	13 (6–19)	16.5 (6–21)	0.85**
Maximum Diameter of RPOC at diagnosis (mm)	26.5 (12–59)	57.5 (5–78)	0.59**

Data are presented as mean ± standard deviation or median (range) or *n* (%). Student’s *t*-test or Mann–Whitney U test was used for continuous variables, and Chi-square test or Fisher’s exact test was used for categorical variables, as appropriate. The specific test used for each variable is indicated in the table.

*Student’s *t*-test,

**Mann-Whitney U test,

***Fisher’s exact test.

The reduction rate in RPOC diameter after treatment initiation was significantly higher in the GnRH antagonist group [50 (0–79.7)% vs. 15.4 (0–56.9)%, *p* < 0.001, Table 3]. UAE was performed if blood loss exceeded 100 ml per hour or in cases of hemorrhagic shock. The frequency of blood transfusions and UAE did not differ between group. However, the proportion of cases requiring surgical intervention was significantly lower in the GnRH antagonist group (30.0%, 6/20) compared to the non-GnRH antagonist group (70.1%, 54/77, *p* = 0.002). Additionally, the time from RPOC disappear to first menstruation after treatment was significantly shorter in the GnRH antagonist group [14.5 (9–71) days] compared to the non-GnRH antagonist group [26.0 (6–95) days, *p* = 0.002] (Table 3).

**TABLE 3:** Clinical courses of gonadotropin-releasing hormone (GnRH) antagonist and non-GnRH antagonist group.

RPOC cases (*n* = 97)	GnRH antagonist (*n* = 20)	Non-GnRH antagonist (*n* = 77)	*P*-value
Blood transfusion (%)	0 (0%)	4 (5.2%)	0.68***
Average reduced length in maximum diameter of RPOC after the initiation of treatment [= before (mm)–after (mm)]	20.5 (0–47)	8.5 (0–33)	0.04**
Average reduction rate in maximum diameter of RPOC after the initiation of treatment [= before (mm)–after (mm)/before (mm)]	50 (0–79.7)%	15.4 (0–56.9)%	< 0.001**
Period from disappear of RPOC to the resumption of the first menstruation after treatment (days)	14.5 (9–71)	26.0 (6–95)	0.002**
Surgical procedures	6 (30%)	54 (70.1%)	0.002***
Trans cervical resection	6 (30%)	33 (42.9%)	0.10***
Dilation and curettage	0 (0%)	9 (11.7%)	0.84***
Hysterectomy	0 (0%)	2 (2.6%)	0.67***
Blood loss (ml)	10 (10–150)	10 (10–160)	0.79**
Uterine artery embolization	0 (0%)	8 (10.4%)	0.29***
Length of hospital stay (days)	3.0 (0–0)	3.5 (2–9)	0.93**

Data are presented as median (range) or *n* (%). Student’s *t*-test or Mann–Whitney U test was used for continuous variables, and Chi-square test or Fisher’s exact test was used for categorical variables, as appropriate. The specific test used for each variable is indicated in the table.

**Mann-Whitney U test,

***Fisher’s exact test.

A multivariate analysis identified that taking oral GnRH antagonists significantly reduced the risk of surgical intervention [adjusted odds ratio (95% confidence interval): 0.20 (0.06–0.58), *p* = 0.003, Table 4].

**TABLE 4:** Multivariate logistic regression analysis of clinical factors associated with surgical intervention: results from models 1–3.

Clinical factors	Model 1 adjusted-OR (95% CI)	*P*-value	Model 2 adjusted-OR (95% CI)	*P*-value	Model 3 adjusted-OR (95% CI)	*P*-value
Age	1.02 (0.94–1.10)	0.98	1.02 (0.94–1.10)	0.662	1.00 (0.93–1.09)	0.917
Assisted reproductive technology	1.23 (0.51–3.05)	0.648	1.19 (0.48–2.98)	0.707	1.06 (0.42–2.7)	0.903
GnRH antagonist	0.20 (0.06–0.57)	0.002	0.23 (0.06–0.54)	0.002	0.20 (0.06–0.58)	0.003
RPOC size	–	–	1.01 (0.99–1.04)	0.299	1.01 (0.99–1.04)	0.341
Body mass index	–	–	–	–	1.04 (0.91–1.20)	0.527
Surgery for abortion	–	–	–	–	1.84 (0.71–5.07)	0.215

*P*-values < 0.05 are considered statistically significant. Adjusted-OR (95% CI): adjusted- odds ratio (95% confidential interval). Retained products of conception (RPOC) size shows the maximum diameter of retained products of conception after the initiation of treatment.

In addition, we performed a more detailed subgroup analysis stratified by age (< 35 vs. ≥ 35 years) to explore differences in clinical outcomes according to GnRH antagonist us. In both age groups, the GnRH antagonist group had a significantly lower surgical intervention compared to the non-use group. Also, the reduction rate in RPOC size was significantly higher in the GnRH antagonist compared to non-GnRH antagonist group (Supplementary Data’s 1a, b). These results suggest that the beneficial effects of GnRH antagonist therapy are generally consistent across different age groups, although certain clinical parameters, such as RPOC shrinkage, may vary by age.

Furthermore, to assess the robustness of our overall findings, we conducted a sensitivity analysis stratified by both ART status and age group (Supplementary Data 2). There was no significant interaction between ART use, age category, and surgical intervention rates, thereby supporting the consistency of the treatment effect across various subgroups. Among the GnRH antagonist group, a comparison between the six cases requiring surgical intervention (surgical intervention group) and the 14 cases that did not (non-surgical intervention group) showed no difference in RPOC diameter at diagnosis. However, the non-surgical intervention group had a significantly higher average reduction rate in RPOC diameter after GnRH antagonist administration [58.0 (5–100)%] compared to the surgical intervention group [8.7 (0–100)%, *p* = 0.03, Table 5).

**TABLE 5:** Patient backgrounds and clinical courses of surgical and non-surgical intervention group in the cases with Gonadotropin-Releasing Hormone (GnRH) antagonist.

GnRH antagonist cases (*n* = 20)	Surgical intervention group (*n* = 6)	Non-surgical intervention group (*n* = 14)	*P*-value
Age (years)	35.3 ± 3.0	33.4 ± 7.2	0.54*
Body mass index (kg/m^2^)	20.8 ± 4.4	22.6 ± 4.3	0.39*
Gravidity	2.5 (1–3)	1.5 (1–4)	0.97**
Parity	1 (0–2)	0 (0–3)	0.76**
Assisted reproductive technology	5 (83.3%)	7 (50%)	0.18***
Average reduced length in diameter of RPOC after treatment [= before (mm)–after (mm)]	2 (0–12)	24.5 (1–24)	0.04**
Average reduction rate in maximum diameter of RPOC after the initiation of treatment [= before (mm)–after (mm)/before (mm)]	8.7 (0–100)%	58.0 (5–100)%	0.03**
Uterine artery embolization	0 (0%)	0 (0%)	NA
Blood transfusion	0 (0%)	0 (0%)	NA
Period from the diagnosis of RPOC to the resumption of menstruation after the end of treatment (days)	75.5 (35–149)	85.5 (21–183)	0.049**

Student’s *t*-test or Mann–Whitney U test was used for continuous variables, and Chi-square test or Fisher’s exact test was used for categorical variables, as appropriate. The specific test used for each variable is indicated in the table.

*Student’s *t*-test: data are presented as mean ± SD or *n* (%).

**Mann-Whitney U test: data are presented as median (range).

***Fisher’s exact test.

## Discussion

This study is the first to evaluate the efficacy of oral GnRH antagonists in treating RPOC. The use of GnRH antagonists significantly reduced the need for surgical intervention compared to conventional management. Goda et al. reported that GnRH antagonists were effective in treating placental polyps, with menstruation resuming 1 month after treatment (14). In our study, all cases treated with GnRH antagonists showed reduced or disappeared blood flow to RPOC, along with a decrease in RPOC size. This suggests that, in addition to reducing uterine arteries flow, blood supply to the intrauterine tissue is cut off and itself may shrinks. These findings suggest that oral GnRH antagonists may help reduce the risk of bleeding and hemorrhagic events, thus decreasing the need for surgical intervention. Since estrogen receptors are expressed in uterine arteries (12), oral GnRH antagonists are expected to act directly on GnRH receptors, leading to a decrease in estradiol (E2) levels and a subsequent reduction in uterine blood flow.

Although direct hemodynamic evidence for GnRH antagonists remains limited, studies of GnRH agonists—which also ultimately lower estrogen levels—have shown increases in uterine artery resistance index (RI), indicating reduced uterine perfusion (15). Thus, further studies are warranted to directly investigate whether similar hemodynamic changes occur with GnRH antagonists must be examined in the future. Additionally, recent research suggests that GnRH antagonists suppress the proliferative capacity of endometrial stromal cells by downregulating c-kit receptor expression (16). This antiproliferative effect may also contribute to the spontaneous regression of RPOC tissue, although the detailed molecular mechanisms remain to be elucidated. In any case, these data dictate that oral GnRH antagonist therapy may contribute to reducing the risk of bleeding and hemorrhagic events. And, in the GnRH antagonist group, no cases required blood transfusion or IVR procedures. This suggests that if surgery was necessary, the GnRH antagonist would have contributed to a favorable perioperative outcome by lowering the risk of preoperative bleeding. Ito et al. reported the usefulness of preoperative administration of GnRH antagonists for transcervical resection myomectomy, as they thin the endometrium and provide a clearer operative field (17). This improved visibility may reduce the risk of intraoperative and postoperative complications, such as endometrial damage and persistent RPOC, suggesting that oral GnRH antagonists may be beneficial if surgery is required (18, 19). Additionally, the shorter time from RPOC diagnosis to menstruation resumption in the GnRH antagonist group may be due to the drug’s ability to reduce blood flow and shrink RPOC size, benefiting patients eager to resume fertility treatment promptly. Interestingly, the age-stratified subgroup analysis revealed that GnRH antagonist use was associated with a lower surgical intervention rate in both age groups (< 35 and ≥ 35 years). This suggests that the beneficial effect of GnRH antagonists in reducing the need for surgery may be consistent regardless of age.

At this stage, it is unclear whether GnRH antagonists are the optimal treatment for RPOC management, however, some advantages may exist when compared to other therapies. GnRH agonists have been reported to reduce uterine blood flow (15); however, they can cause a transient flare-up phenomenon characterized by an initial rise in estrogen levels, which may provoke bleeding. In contrast, GnRH antagonists rapidly suppress estrogen without causing a flare-up, potentially allowing for safer use. In the present study, no cases of massive bleeding were observed following GnRH antagonist administration, further supporting its safety profile. Compared to progestin-based therapies, which modulate the endometrium indirectly, GnRH antagonists exert a direct estrogen-suppressive effect, which may enhance their efficacy in promoting RPOC regression. When conservative management fails, surgical intervention becomes necessary; in such cases, preoperative reduction of blood flow may decrease intraoperative bleeding, improve surgical field visibility, minimize endometrial injury, and facilitate complete lesion resection. Although UAE offers strong hemostatic efficacy, it has been associated with postoperative intrauterine adhesions and an increased risk of miscarriage (20). Thus, when future fertility is a priority, minimally invasive pharmacological therapies such as GnRH antagonist treatment may represent a preferable alternative. However, it is important to note that GnRH antagonists are not currently approved for the treatment of RPOC and entail considerable costs. Therefore, careful patient selection, thorough counseling, and informed consent are essential. Further accumulation of clinical data is needed to confirm their efficacy and safety. Although the benefits of GnRH antagonists may be promising, we must not forget the risk of massive bleeding that RPOCs still carry. When managing patients on GnRH antagonists in an outpatient setting, educating them about the potential need for emergency interventions, such as UAE, is essential. Timely collaboration with the radiology department for outpatient management should also be ensured.

This study has several limitations. We restricted the study population to cases treated after 2014, when surgical indications at our institution became more standardized, and compared baseline characteristics between the GnRH antagonist group and the historical control group. Multivariate logistic regression analyses adjusting for major confounders were also performed; however, the possibility of bias inherent in using historical controls cannot be entirely excluded. Moreover, although subgroup analyses stratified by age and sensitivity analyses stratified by both ART status and age generally supported the consistency of the treatment effect, these additional analyses could not completely eliminate the potential for bias. Future research should involve prospective studies using contemporaneous control groups and standardized protocols to more accurately validate these findings. And, we hypothesize that the reason of decrease of RPOC size reduction is due to reduced blood flow by an oral GnRH antagonist, but more detailed assessment of blood flow and analysis of other factors is now needed to investigate the exact reduction mechanism. Additionally, despite the promising results, GnRH antagonists are not yet covered by insurance for RPOC treatment. A larger number of cases is needed to accurately evaluate their effectiveness. Future studies should compare treatments within the same time frame, use standardized protocols, and increase the sample size to better determine the optimal administration method. Some cases in the GnRH antagonist group may have resolved spontaneously, highlighting the need for further investigation to avoid overtreatment.

## Conclusion

This study highlighted the potential of oral GnRH antagonists in treating RPOC. Future research should focus on identifying appropriate indications and refining administration methods. Informed consent and thorough patient education are essential. Larger studies are needed to confirm these findings and assess pregnancy outcomes in treated cases.

## Data Availability

The original contributions presented in this study are included in this article/Supplementary material, further inquiries can be directed to the corresponding author.
